# The Distributional Characteristics of Heavy Metal in Jiangsu Province Shoal Sea

**DOI:** 10.1155/2013/142065

**Published:** 2013-05-08

**Authors:** Yu Wenjin, Zou Xinqing

**Affiliations:** ^1^Key Laboratory of Meteorological Disaster of Ministry of Education, Nanjing University of Information & Technology, Nanjing 210044, China; ^2^Hohai University, Key Laboratory of Coastal Disaster, Nanjing 210093, China; ^3^Ministry of Education Key Laboratory for Coast and Islands Development, Nanjing University, Nanjing 210093, China

## Abstract

After the analysis of surface samples and core samples collected in Xinyanggang tidal land, the contents of Pb, Cu, Zn, and Cr were obtained and analyzed in this paper. The heavy metal accumulation rule and pollution status were studied by Index of geo-accumulation, latent ecological risk index method, and elements accumulation index method. The research suggests that (1) the contents of heavy metal Pb, Cu, Zn, and Cr in Xinyanggang tidal land have the same change trend, and such trend remains unchanged after the data were normalized, while the fluctuation range becomes smaller. (2) After analyzing the heavy metal content in the surface samples, it was revealed that the contents of heavy metals are getting lower from high tidal zone to low tidal zone, but the ranges of the change were different. Cu, Ni, and Zn emerge obvious decline from supratidal zone to subtidal zone, while the changes of Cr and Pb are not obvious. (3) Pb and Cr contents in Xinyanggang tidal land present accumulative character, as Pb in Xinyanggang is 3 times as much as the local background value, whose EF reaches 3.774. (4) RI value in Xinyanggang is 23.552, which indicates that though Xinyanggang tidal land has some heavy metal pollution and accumulation, there are no ecosystem risks, and the whole Xinyanggang core area environment quality is relatively good.

## 1. Introduction

As a special coastal wetland, tidal wetland is a complicated multiple-functional ecosystem with unique ecovalue and resource potential. In tidal zone, heavy metals cannot be purified through water self-purification; they normally sink into sediments after deposition and accumulation in tidal land through complicated physical, chemical, and biological process. Xu carried out researches on the spatial distributions of heavy metals and their dynamic accumulating characteristics in Shanghai coastal tidal land [1] which indicate that, in large scale, the spatial distribution pattern of the heavy metal Cu, Zn, Cr, and Pb in tidal flat sediments was not directly affected by coastal pollution discharges, rather they were closely related to sediment dynamics; while in small scale, the content of heavy metals in the tidal land near the drain was closely related to the drain. Bi Ch [2] studied different variation patterns of the existing form of heavy metal Cu, Zn, Cr, and Pb in different seasons, different locations in tidal land near the Bailonggang drain, and the shape, content, and distribution in the root sediments, finding that the contents of heavy metals in the tidal land near the Bailonggang drain were all obviously above the background value in the Shanghai tidal flat and that the sewage input had some impact on the existing form of heavy metals in sediments. 

Northern-Jiangsu tidal wetland, which is a typical muddy tidal flat wetland, has the greatest area, the maximum ecosystem types, and the most complicated erosion-accumulation changes in China, even in the world. It was formed by the sediments from the ancient Yellow River Delta and Changjiang River Delta, which were washed by wave collisions and tides in the Yellow Sea and the East China Sea. With the length of 444 km, Jiangsu coastal wetland has the area of 5,100 km^2^. Within this area, there are abundant river and marsh developed. The ecosystem in the core area of Yancheng Reserve remains in natural status, with limited human impacts. Therefore through analysis of tidal flat surface samples and core samples in the core area of the Reserve and the contents of heavy metal Cu, Zn, Cr, and Pb in different geomorphologies and different vegetation conditions and upon verifying between indicators, the following works had been done, that the distribution pattern of the above indicators was discussed, the biogeochemical process in different vegetations and sediments was analyzed, and the roles of different tidal flat geomorphologies and their vegetations in the enrichment of the heavy metals in the whole tidal wetland were compared.

## 2. Materials and Methods

### 2.1. The Collection of Samples and Disposal

**Figure 1:**
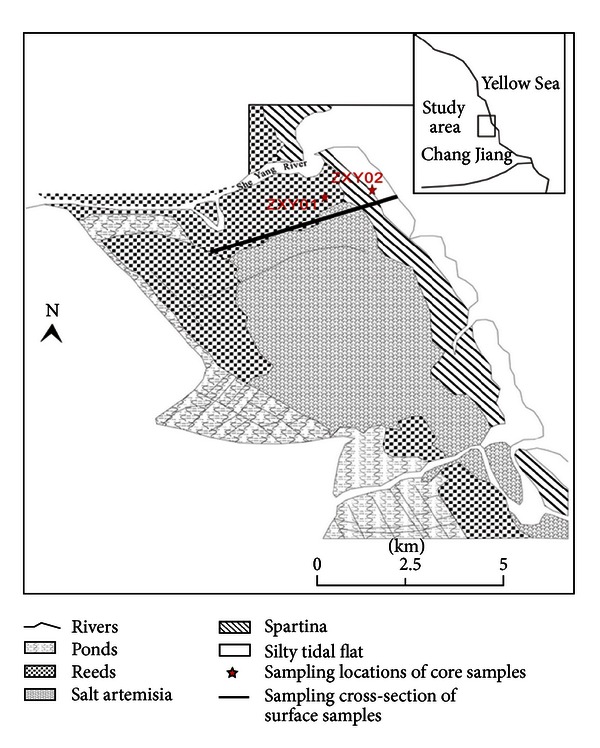
Map of sampling locations.

Between August 17 and 19 2005, two cores (SY01, SY02) were collected in the core area of Yancheng red-crown crane National Natural Reserve (Xinyanggang in Sheyang County); surface samples were collected every 20 m along the cross-section from the west to the east, 220 samples in all, the sampling locations refer to Figure 1. Two cores were collected using 70 mm inner diameter and 75 mm outer diameter PVC pipes which directly access into the ground. It was pointed by the hand GPS, precision 10 m. After sealing at field, we brought them to the lab, took out the samples, dissected, took photos, and described the lithologic characters and deposition configurations. We conserved one-half of the dissected core samples and the other half was divided every 2 cm, 72 parts in total, using freeze-drying machine ALPHA-1-4 produced by the German Martin Christ Company to hypothermia lyophilize samples. For the computation method of water content and volume, refer to Ren M E, 1983. The grain size of the samples was analyzed using the Laser Particle Size Analyzer Mastersizer 2000 produced by Britain, accomplished in the Ministry of Education Key Laboratory For Coast and Islands Development, Nanjing University. The grain size parameters computation used the Moment method.

We took 260 lyophilized samples (including all the surface samples, core samples choosing 0–50 cm, and 50–100 cm every other sample), removed plant debris and stones, ground and passed then through 100 mesh sieve, preserved then in plastic bottles, and then kept then in dryer in order to determine the content of heavy metals. The heavy metal tests were carried out in ICP-MS Heavy Metal Analysis Room of the State Key Laboratory for Mineral Deposits Research, Nanjing University. The process is as follows: weigh 0.5 g sample accurately, then resolve it using HNO_3_-HCL-HCLO_4_ acid in the triangular flask, slake it in the 2040 programmable slacker, and determine the volume, and determine the content of Cu, Zn, Pb, Ni, Fe, Cr, Fe, Li, Al, and other heavy metals in soil using ICP-MS.

The instruments used are the following: Orient MDS-9000 Microwave Slaking System (Xi'an Aoruite Technology Development Corporation); HP4500series300 Plasma Mass Spectrometer (Hewlett-Packard); Ultra-pure Water NG (MLLi-Q) manufactured by American MLLipore Company. All containers were soaked overnight using 20% hydrogen nitrate and rinsed with water three times.

### 2.2. Data Handling and Methods

The map of sampling locations and a part of figures and tables were made using Mapinfo 7.0 and Coreldraw 10; most figures and data statistical analysis were accomplished using excel 2000 and Origin 6.0, and part of the data was handled and statistically analyzed with SPSS10. and with SPSS10. The correlation analysis between heavy metals and the correlation analysis between heavy metals and grain size were also carried out.

### 2.3. The Heavy Metal Pollution Evaluation Method 

#### 2.3.1. Index of Geoaccumulation

In order to evaluate the enrichment conditions and pollution conditions of heavy metals in the core area, the Index of geoaccumulation, *I*_geo_, presented by German scientist, Muller, was adopted [3], which was used to quantitatively analyze heavy metal pollution in fluids. Its equation can be seen as follows:

(1) $$\begin{array}{c} I_{\text{geo}}=\text{lo}{\text{g}}_{2}\left(\frac{C_{n}}{AB_{n}}\right). \end{array}$$

In the above equation, *C*_*n*_ is the content of the *n* element in the sediment, *B*_*n*_ is the geochemical background value of the bedrock, and *A* is the constant for modifying the fluctuation of the background value caused by lithogenous movement, usually 1.5. According to this method, the heavy metal pollution in the sediments was divided into 7 degrees, as can be seen in Table 1. 

#### 2.3.2. Heavy Metal Latent Ecological Risk Assessing Method

In this paper, the Latent ecological index method put forward by the Sweden scientist Hakanson was adopted [4], which is a method for assessing heavy metals in soils or in the sediments from the perspective of sedimentation based on the nature and the environmental behavior characteristics of heavy metal. This method not only considers the heavy metal content in soils, but also links the ecological effects of heavy metals, environmental effects, and toxicological effects together and adopts the comparable, equivalent attribute Index classification method for evaluation. Potential ecological index correlates to the individual pollution coefficient, heavy metal toxicity coefficient, and potential ecological risk individual coefficient; its equation can be seen as follows:

(2) $$\begin{array}{c} \text{RI}=\sum E_{r}^{i},\\ E_{r}^{i}=T_{r}^{t}\times C_{f}^{i},\\ C_{f}^{i}=\frac{C_{0}^{i}}{C_{n}^{i}}. \end{array}$$

In the above equations, *E*_*r*_^*i*^ is the potential ecological risk individual coefficient, and *T*_*r*_^*i*^ is the heavy metal toxicity; its corresponding coefficient adopts heavy metal toxicity standardized coefficient developed by Hakanson as evaluation basis; the heavy metal toxicity level priority is Pb = Cu > Zn, and the toxicity coefficient is Hg: 40, Cd: 30, As: 10, Pb: 5, Cu: 5,Cr: 2, Zn: 1. *C*_*f*_^*i*^ is the individual pollution coefficient, *C*_*n*_^*i*^ is the reference value, and *C*_0_^*f*^ the measured value of the surface heavy metal in soil. The heavy metal individual pollution coefficient can refer to Aloupi and Angelidis [5], and the heavy metal pollution ecological risk coefficient and the ecological risk index can be seen in Table 2.

#### 2.3.3. Sediment Enrichment Factor Method of Heavy Metals

Using the *K*_SEF_, sediment enrichment factor method to evaluate the degree of the heavy metal pollution is developed by Kemp [6], and its equation is

(3) $$\begin{array}{c} K_{\text{SEF}}=\frac{(S_{E}/S_{\text{Al}}-a_{E}/a_{\text{Al}})}{\left(a_{E}/a_{\text{Al}}\right)}. \end{array}$$

In the above equation, *K*_SEF_ is the heavy metal enrichment factor in sediments, *S*_*E*_ is the content of heavy metals in sediments, *S*_Al_ is the Al content in sediments, *a*_*E*_ is the heavy metal content in uncontaminated sediments, and *a*_Al_ is content of Al in the uncontaminated sediments. Al was chosen to be the reference element due to its inertness in its migration. In Xingyanggang Al could be replaced by Li. In sediments the greater *K*_SEF_ is, the heavier the heavy metal pollution can be. If *K*_SEF_ > 0, it means that there is heavy metal enrichment, and the enrichment level can be signified by its value. Using *K*_SEF_ method for heavy metal geochemical correction in sediments not only can remove the impact of particle size, but also can avoid impact of the secondary nature of sediments on the measured values. In order to reflect the enrichment of the heavy metals, the enrichment factor equation was introduced as following:

(4) $$\begin{array}{c} EF=\frac{M_{0}/\text{L}{\text{i}}_{0}}{M_{B}/\text{L}{\text{i}}_{B}}. \end{array}$$

In the above equation, *M*_0_ relates to heavy metals content in the tidal flat surface, *M*_*B*_ is the background value of the heavy metal, and EF means enrichment factor. Li_0_ means the content of Li in the tidal flat sediments, and Li_*B*_ means the background value of Li. The enrichment factor classification standard can be shown in Table 3. 

## 3. Results and Discussions 

### 3.1. The Vertical Variation Pattern of Heavy Metals

After the study on the particle size and the vertical variation pattern of the heavy metals of the core ZXY01, ZXY02, it could be found that (1) the tendency of heavy metal Zn, Pb, and Cu in ZXY01 was basically the same; on the whole it decreased fluctuatingly from the surface to the downward, at −15 cm, −30 cm, and −55 cm, it formed the peak value, while at −25 cm, −55 cm it formed the valley value; (Pb was noticeably increasing only bellow −60 cm, different from the other two metals); the peak and valley values of them were in good coordination. The contents of Fe, Ca, and Mn were fluctuatingly increasing; at −25 cm and −55 cm it formed the peak value, so they were synchronous (refer to Figure 3). Ni, Cr, and Mg had no obvious increasing tendency; they fluctuated in a certain scope but formed peak value at −25 cm and −55 cm all the same. (2) After using Li as the standard normalized element, the fluctuation range of normalized heavy metals was all significantly narrower than that of the heavy metal content, with the former peaks and troughs lagging behind the latter. The basic fluctuation trend did not change, while the trend of Pb/Li opposed to that of Pb content. Obviously, Pb displayed different distribution characteristics from that of other elements. Except that the content of heavy metals Zn, Pb of ZXY02 fluctuated with depth in a certain range, the remaining heavy metals shown in Figure 3 increased with the depth. After being normalized, the fluctuation scope of heavy metals significantly narrowed, and the fluctuation trends of the elements were similar to that of the contents of heavy metals with the exception of Pb. (3) Compared with ZXY01, the normalized value of ZXY02 was relatively more stable. In these two cores, normalized Pb displayed different characteristics from the other elements, indicating that the sources of Pb and other elements were different, and some Pb pollution might also exist in the core. 

**Figure 2:**
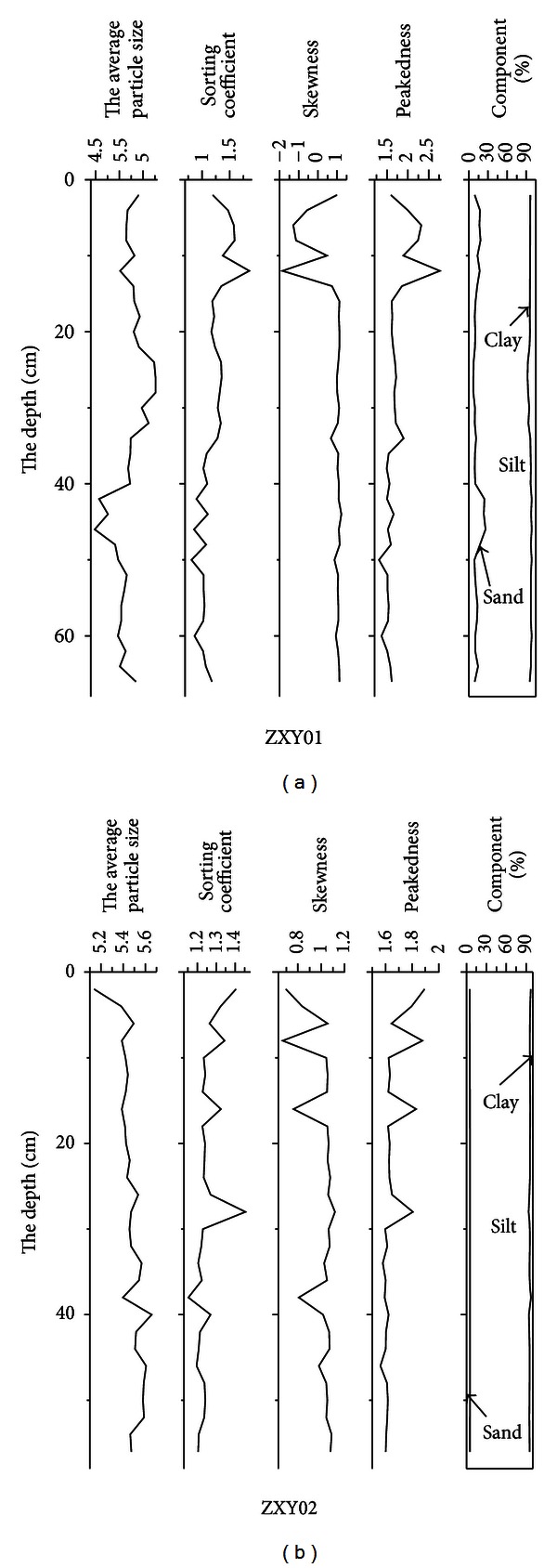
Grain size character diagram of cores ZXY01 and ZXY02 in the Xinyanggang tidal flat.

**Figure 3:**
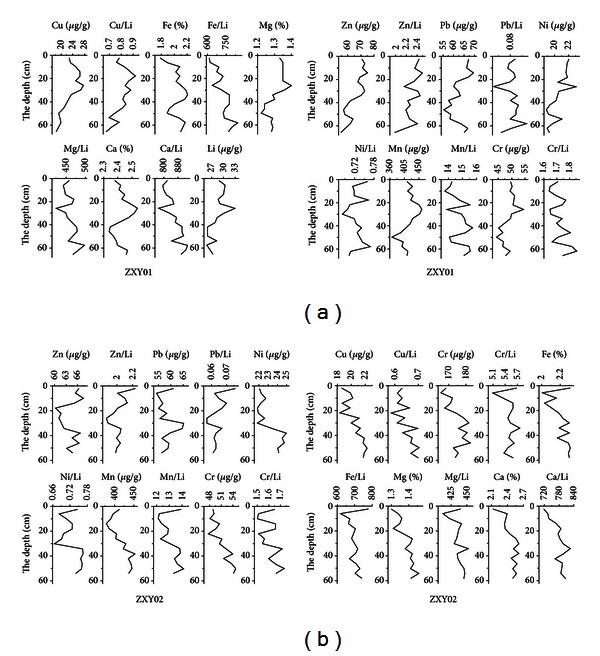
Metal content change map of Xinyanggang pillars ZXY01 and ZXY01.

When comparing grain size of ZXY01 with ZXY02, the content of clay and sand of ZXY01 was more than that of ZXY02, and the change from top to bottom was greater. The silt content of ZXY02 was higher than that of the other, while the content of clay and sand from the top to the bottom was relatively stable (Figure 2). Compared with the most heavy metal deposition pattern in North Jiangsu, the heavy metal deposition was mainly controlled by clay content and material source in the sediment and was positively correlated with clay content. This was the reason why the content of Cu and Zn of ZXY02 was less than that of ZXY01 and the changes were more stable (Figure 3). Only the Pb content change had unique accumulating law, and it could be deduced that human activity contributed a great deal to the accumulation of Pb in this area. 

### 3.2. The Horizontal Distribution Pattern of Heavy Metals

The heavy metal content distribution characteristics of the horizontal section show the decrement trend from high tide zone to low tide zone, but the decrement speed varied. As observed the content of Cu, Ni, and Zn declined more notably from the upsurge to the low ebb, while Cr, Pb had no obvious decreasing trend, showing that the content of Cr, Pb in tidal flat sediments was not significantly affected by water dynamic. Particularly Pb gas deposits accounted for larger proportion. Seeing from fluctuation amplitude, Zn, Pb had larger fluctuation amplitude, respectively, 79.19~63.92 (*μ*g/g) and 71.54~58.7 (*μ*g/g), and the other elements had smaller fluctuation amplitude, copper: 29.98~24.07 (*μ*g/g), Ni: 25.08~19.52 (*μ*g/g), and Cr: 58.31~48.79 (*μ*g/g). As can be seen from Table 4, the content change scope of surface heavy metals of Xingyanggang core area was smaller than that of Sheyang County in comparison, so it could be seen that overall protective effect was better in the core area. 

## 4. Heavy Metal Pollution Evaluation

### 4.1. The Present Heavy Metal Pollution Analysis

After analyzing 220 surface samples, in general, the content of the chemical elements of Xingyanggang tidal wetlands in the surface sediments was in the following order: Mn>Pb>Zn>V>Cr>Cu>Li>Ni. In this paper, representative elements Cu, Pb, Zn, and Cr were used to evaluate tidal flat heavy metal pollution as indicators, which could indicate the human activity impacts on the tidal flats. Many researchers use the average value of shale presented by Turekian and Wedepohl [7] or the average crustal abundance value presented by Taylor [8] as a reference background value. However, in different regions due to different sediment sources, background values should be chosen which had comparable mineral compositions with contaminated sediments and had unpolluted sediment elements value, namely, from the deepest core sites with the absence of bioturbation, so the trace elements prior to the industrialization activities were estimated to be background values [5] Xingyanggang region was a traditional agricultural area, the core area protected well, and no large-scale development conducted, basically in pristine natural state. Throughout Sheyang County industrialization just started in the mid-1980s. Considering that the Xingyanggang tidal flat material sources come from the Old Yellow River sediments, the Yangtze River sediments, and Jiangsu radial sand ridges, in this paper, the soil reference value adopted the heavy metal Pb, Cu, Zu, and Cr average background value of Yangtze River estuary northern shore tidal flat and eastern China tidal flat as the value [9] (see Table 5). For classification standards of heavy metal individual pollution coefficient, one can refer to (Editor-in-chief by Nanjing Institute of Soil, CAS, 1978).

The research reveals that contents of elements Pb, Cr in Xinyanggang intertidal sediments are different from those in Yangtze River mouth sediments, Yellow River Mouth sediments, China shallow sea sediments, and Jiangsu soil and showed difference of enrichment level (Table 4). According to China's marine sediment quality standard, Pb exceeded sediment standard value of the bottom (50 *μ*g/g). Cu, Cr, and Pb in Xinyanggang tidal flat were all beyond the background values, in which Pb is the worst, with the EF value reaching 3.774, which was three times higher than the background values of China east coast. Meanwhile Pb enrichment degree in Xinyanggang tidal flat reaches the strong enrichment scope (Table 5). In conclusion, Pb pollution is significant. *K*_SEF_ values of Cu, Cr, and Pb are greater than zero and had a certain degree of enrichment. Pb is more significant, corresponding to its EF value. From EF and *K*_SEF_ value, Zn had no enrichment, Pb had significant enrichment, and Cr, Cu had slight enrichment. Cu's EF value reaches 1.173, exceeding China east coast background value but less than that of north bank of Yangtze River estuary. EF value of Cr reaches 1.326, lower than the background value of China east coast but higher than that of north bank of Yangtze River estuary. In other words, the enrichment degrees of Cu and Cr are close to the background values, with no significant pollution deposition occured. Because of the industry developed in this region, Pb's pollution is significant.

From *I*_geo_ value, Xinyanggang Pb pollution reaches partial pollution degree, while Zn, Cr, Cu, and Li are not polluting, corresponding to the former research results in the preamble. 

### 4.2. The Vertical Change of Heavy Metal Pollution

**Figure 4:**
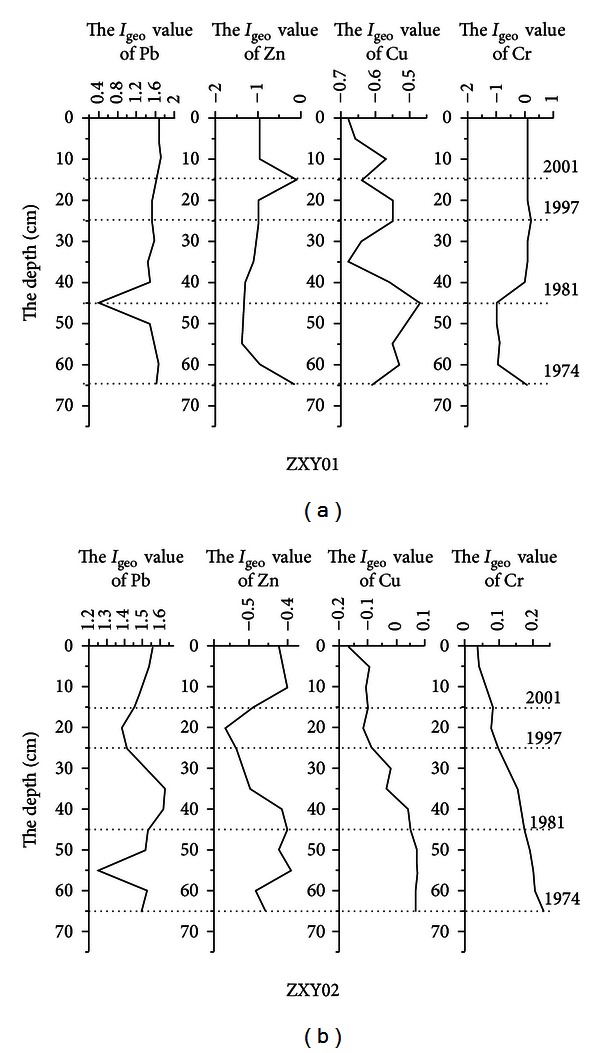
Vertical change process of heavy metal pollution in Xingyanggang tidal flat.

According to ^210^Pb dating method, the average sedimentary rate of Xinyanggang tidal flat was 2.85/a, so that heavy metal pollution change process in the last 25 years can be obtained from the study of core samples (Figure 4). It is showed by ZXY01 and ZXY02 that since 2001, *I*_geo_ value of Pb in Xinyanggang is above 1, indicating middle plus level pollution. From 1974 to 1981, Pb pollution was on the downward trend, which was because lead content in fuel was controlled by law. From 1981 to 1997, Pb pollution fluctuated in the low level; after 1997, Pb pollution was on an upward trend, which was related to the development of Sheyang chemical and metallurgical industries, and so forth [10]. Thus, it was proposed that a buffer zone should be set up between the core area and the development zone to better protect the core area environment. The research showed that no significant Cu, Zn pollution occurred in the tidal flat of Xinyanggang core zone, and Cu, Zn pollution was on the downward trend. Although there was minor contamination of Cr (–1–0.2), it was only a lighter extent and on a downward trend. This showed that since the nature Reserve establishment in the core area in recent years, good effects have achieved. 

### 4.3. The Potential Ecological Harm Analysis of Heavy Metals

Upon study, it was found that there were a certain enrichment and pollution of heavy metal Pb, Cr in Xinyanggang core area. In order to find out the heavy metal impact in the core area on the national protection animal, the red-crowned crane, and on the ecological environment pollution, the potential ecological index method was adopted which was presented by Swedish scientist Hakanson, which is a method for assessing heavy metals in soils or in the sediments from the perspective of sedimentation based on the heavy metal nature and its environmental behavior characteristics. This method not only considers the content of heavy metals in soil, but also links the ecological effects of heavy metals, environmental effects and toxicological effects together. The results showed that (see Table 6) Pb, Zn, Cu, and Cr had no ecological pollution, Pb had the highest indexes, and its *E*_*r*_^*i*^ and *C*_*f*_^*i*^ reached 15.635 and 3.127, both in the minor scope of ecological harm. The indicators' order from high to low was Pb, Cr, Cu, and Zn, all in a safe area, and ecological pollution hazards had not appeared yet. RI value was 23.552, far less than light ecological hazard upper limit 150, indicating that although there were some heavy metal enrichment and pollution in the core area, yet no ecological hazards had been produced, and overall environment quality in Xinyanggang core area was excellent. 

## 5. Conclusions 

Through research it could be found that (1) the contents of heavy metal Pb, Cu, Zn, and Cr in Xinyanggang tidal land have the same change trend, and the fluctuation range becomes greater from deep part to surface. After using Li as the standard normalized element, the fluctuation ranges of all heavy metal normalized elements were significantly narrower than that of the heavy metals' content, with the former peaks and troughs lagging behind the latter. The main fluctuation trend keeps unchanged. The change trend of Pb/Li was counter to that of Pb content. (2) From the heavy metal content distribution characteristics of the horizontal section, we can find that they all displayed the decreasing trend from high tide to low tide, but the decreasing speed varied. The contents of Cu, Ni, and Zn declined more notably from the upsurge to the low ebb, while Cr, Pb had no obvious decreasing trend. (3) Cr and Pb in Xinyanggang tidal flat showed different enrichment degree, in which Pb exceeded the standard most, with EF value reaching 3.774, three times as much as the background values of China east coast. Its enrichment degree in Xinyanggang tidal flat reaches the strong enrichment scope, so Pb pollution is significant. (4) Xinyanggang RI value is 23.552, far less than light ecological hazard upper limit 150, indicating that although there are some heavy metal enrichment and pollution in the core area, no ecological hazard has been produced yet, and overall environment quality in Xinyanggang core area was excellent. 

## Figures and Tables

**Table 1:** Index of geoaccumulation and pollution ratings.

Degree	0	1	2	3	4	5	6
*I*_geo_	<0	[0,1]	[1,2]	[2,3]	[3,4]	[4,5]	[5,6]
Pollution degree	No pollution	Light pollution	Partial pollution	Middle pollution	Heavier pollution	Heavy pollution	Serious pollution

**Table 2:** Grade standard of *E*_*f*_^*i*^ and RI.

Pollution coefficient *E*_*r*_^*i*^	Pollution index RI	Pollution level
<40	<150	Light ecological risk
40–79	150–299	Middle ecological risk
80–159	300–600	Strong ecological risk
160–320	>600	Very strong ecological risk
>320		Extremely strong ecological risk

**Table 3:** Enrichment degree of heavy metals.

EF	<0.25	0.25~0.5	0.5~0.75	0.75~1.5	1.5~2	2~4	>4
Enrichment degree	Extremely depletion	Strong depletion	Weak depletion	Proximity enrichment	Weak enrichment	Strong enrichment	Extremely enrichment

**Table 4:** Heavy metal content of surface samples in Xingyanggang tidal flat.

Elements	Surface samples of Sheyang County		Surface samples in Xingyanggang tidal flat	
	Mean value	Variation scope	Mean value	Variation scope
Zn	72.2	79.19~63.92	68.95	73.14~64.02
Pb	65.76	71.54~58.7	65.83	71.88~56.01
Co	14.28	15.37~12.99	14.27	15.53~13.09
Ni	22.96	25.08~19.52	22.52	23.85~21.3
Mn	481	506.6~449.7	443.98	477.1~397.8
Cr	53.6	58.31~48.79	52.83	56.28~48.42
Cu	27.77	29.98~24.07	20.84	22.87~18.14
Li	32.8	36.39~28.87	31.49	33.78~30.05

**Table 5:** Heavy metal content and background values in Xinyanggang tidal land.

Area	Pb (*μ*g/g)	Zn (*μ*g/g)	Cu (*μ*g/g)	Cr (*μ*g/g)	Li
Background value in Xinyanggang tidal flat	21.05	88.5	21.35	46.8	38
Mean value in Xinyanggang tidal flat	65.83	68.95	20.84	52.83	31.49
Maximum value in Xinyanggang tidal flat	71.88	73.14	22.87	56.28	33.78
Minimum value in Xinyanggang tidal flat	56	64.02	18.14	48.42	30.05
Background value in north shore tidal flat of Changjiang estuary	22.1	112	27.7	33.6	—
Background value in eastern China tidal flat	20	65	15	60	38
Marine survey value in Jiangsu tidal flat heavy metal survey in 1986	25	80	30	45.7	
Marine baseline survey value of Dafeng County in 1997	28.06	—	—		
*I*_geo_	1.06	−0.954	−0.62	−0.409	−0.857
Pollution degree	Heavier pollution	No pollution	No pollution	No pollution	No pollution
*K*_SEF_	2.774	−0.06	0.192	0.362	
EF	3.774	0.94	1.178	1.362	
Enrichment degree	Strong enrichment	Proximity enrichment	Proximity enrichment	Proximity enrichment	

**Table 6:** Estimation result of the ecological damage of heavy metals from Xinyanggang tidal flat.

	Tide beach	In the tidal flat	The ebb shoal
*C*_Pb_^*i*^	3.127	3.055	3.190
*C*_Cu_^*i*^	2.108	1.842	1.198
*C*_Zn_^*i*^	1.339	2.069	1.006
*E*_Pb_^*i*^	15.635	22.7775	20.950
*E*_Cu_^*i*^	15.540	11.210	5.990
*E*_Zn_^*i*^	6.439	6.052	2.017
RI	23.552	36.0835	27.946
The evaluation results	Slight ecological risk	Slight ecological risk	Slight ecological risk
